# Supplementary material for: Detection of Fused Genes in Eukaryotic Genomes using Gene deFuser: Analysis of the Tetrahymena thermophila genome
Source: BMC Bioinformatics. 2011 Jul 11;12:279. doi: 10.1186/1471-2105-12-279 (PMC3143110; doi:10.1186/1471-2105-12-279)
Supplement: Additional file 1 — Results of Gene deFuser for the Tetrahymena thermophila genome. This zip file contains the raw results of the analysis of the Tetrahymena genome using Gene deFuser. To view the contents, unzip the file and open the Final_Tet.html file in the resulting folder. [file 1471-2105-12-279-S1.ZIP › Results/Final_Tet.html]

Gene deFuser -- Results of Job Final\_Tet

 


Gene deFuser

| Home | Retrieve Results | References | Help |
| --- | --- | --- | --- |

# RESULTS of JOB Final\_Tet:

Download a zip file with the results.

  
The following 80 genes are candidate fusion genes. For each gene, the segments taken from N- and C-termini of the protein sequences returned NO common ortholog group hits:  
  

**The following 28 candidate fusion proteins have only one COG hit on each terminus:**   
  
1. 3810.m01825  
2. 3729.m00007  
3. 3756.m00246  
4. 183.m00041  
5. 263.m00037  
6. 257.m00032  
7. 3698.m00061  
8. 3676.m00002  
9. 3729.m00010  
10. 224.m00030  
11. 178.m00048  
12. 3692.m00017  
13. 3729.m00011  
14. 3710.m00056  
15. 91.m00111  
16. 349.m00006  
17. 394.m00007  
18. 3699.m00163  
19. 3729.m00012  
20. 3707.m00111  
21. 3733.m00633  
22. 183.m00040  
23. 3729.m00008  
24. 3729.m00013  
25. 3687.m00144  
26. 3748.m00311  
27. 3729.m00772  
28. 3701.m00213  
  
**The following 52 proteins have more than one COG hit on each terminus:**   
  
29. 3823.m01136  
30. 3713.m01023  
31. 112.m00150  
32. 3693.m00110  
33. 22.m00350  
34. 151.m00103  
35. 3681.m00006  
36. 3813.m01641  
37. 3703.m00007  
38. 3707.m00133  
39. 90.m00211  
40. 109.m00143  
41. 115.m00135  
42. 135.m00084  
43. 3829.m01190  
44. 3753.m00398  
45. 3730.m00064  
46. 3721.m00062  
47. 275.m00020  
48. 3699.m00043  
49. 3811.m01052  
50. 3836.m02173  
51. 3692.m00067  
52. 21.m00194  
53. 183.m00043  
54. 273.m00034  
55. 91.m00128  
56. 3714.m00089  
57. 3699.m00126  
58. 3684.m00127  
59. 3717.m00027  
60. 14.m00425  
61. 3729.m00009  
62. 3814.m00878  
63. 3692.m00143  
64. 3674.m00006  
65. 92.m00124  
66. 3673.m00016  
67. 3813.m01638  
68. 3709.m00022  
69. 219.m00028  
70. 91.m00132  
71. 3700.m00044  
72. 3824.m02978  
73. 188.m00073  
74. 3698.m00018  
75. 3794.m00173  
76. 3813.m01443  
77. 3687.m00148  
78. 3746.m00310  
79. 3729.m00066  
80. 3733.m00096

Contact: Andre Cavalcanti\_\_\_\_\_Last Modified September 14, 2010
